# A Multifunctional Sensor in Ternary Solution Using Canonical Correlations for Variable Links Assessment

**DOI:** 10.3390/s16101661

**Published:** 2016-10-21

**Authors:** Dan Liu, Qisong Wang, Xin Liu, Ruixin Niu, Yan Zhang, Jinwei Sun

**Affiliations:** 1 Harbin Institute of Technology, School of Electrical Engineering & Automation, Harbin 150001, China; zyhit@hit.edu.cn (Y.Z.); jwsun@hit.edu.cn (J.S.); 2 Harbin Institute of Technology, School of Transportation Science and Engineering, Harbin 150090, China; xinliu@hit.edu.cn; 3 Department of Electrical and Computer Engineering, Virginia Commonwealth University, Richmond, VA 23284, USA; rniu@vcu.edu

**Keywords:** multifunctional sensor, ternary solution, content measurement, canonical correlation analysis

## Abstract

Accurately measuring the oil content and salt content of crude oil is very important for both estimating oil reserves and predicting the lifetime of an oil well. There are some problems with the current methods such as high cost, low precision, and difficulties in operation. To solve these problems, we present a multifunctional sensor, which applies, respectively, conductivity method and ultrasound method to measure the contents of oil, water, and salt. Based on cross sensitivity theory, these two transducers are ideally integrated for simplifying the structure. A concentration test of ternary solutions is carried out to testify its effectiveness, and then Canonical Correlation Analysis is applied to evaluate the data. From the perspective of statistics, the sensor inputs, for instance, oil concentration, salt concentration, and temperature, are closely related to its outputs including output voltage and time of flight of ultrasound wave, which further identify the correctness of the sensing theory and the feasibility of the integrated design. Combined with reconstruction algorithms, the sensor can realize the content measurement of the solution precisely. The potential development of the proposed sensor and method in the aspect of online test for crude oil is of important reference and practical value.

## 1. Introduction

According to the theory of hydrocarbon generation, extracted crude oil usually contains amount of salt and water because the thickly oil-covered earth always appears along with salt and water layer [1]. Most of the salts are inorganic and water-soluble, and are mainly the chloride of the alkaline-earth metals. Water in oil exists in three different states: suspended, emulsified, and dissolved [2]. The presence of salt and water affect crude oil mining, refining, and marketing directly. This is the reason that crude oil must be dehydrated from the oil field before its transmission. Even so, the oil in the refinery still contains certain amounts of salt and water [3]. As a matter of fact, crude oil mixed with too much water could increase heating consumption and lead to instability in the procedure of distillation. The salt in the crude may also cause damage by generating highly corrosive hydrochloric acid. Furthermore, salts may deposit and form salt fouling on the pipe wall, which can not only reduce the thermal efficiency, but also increase the flow resistance and even block the pipe [4]. In addition, catalyst poisoning sometimes appears due to the existence of water and salt. Consequently, before being fed into refining machine, crude oil has to be dehydrated and desalinated. Accurate and timely measurement of moisture content and salt content is one of the important requirements in processes such as oil production, storage, transportation and refining. It is of great significance for locating the oil layer, estimating the output, predicting the life of oil well, improving the transportation method and developing the refining technology. Meanwhile, reliable data on water and salt concentrations may give an indication of the working status of the oil well. It also helps reduce energy consumption and realize efficient management of oil field [5].

At present, there are many methods to measure salt concentration in crude oil, among which the most representative ones are microcoulometry [6], conductometry [7,8], and potentiometric titration [9]. Microcoulometry is a time-consuming and inconvenient method. It can be affected by external factors easily because of the tedious operation and the strict requirement of experiment. Conductometry is one of the most popular methods and is currently used in evaluating the effects of flow pattern and salinity of oil-water two-phase flow on water holdup measurement [8]. However, it is mostly applied to the extract of crude sample for offline measurement. This shortcoming makes it available only for laboratory conditions and restricts the extensibility greatly. As for potentiometric titration, the titrimetric efficiency depends on the quality of electrode and is highly demanding for operators’ experience. Thus, it is only suitable for experienced professionals.

Similarly, measurement methods of oil concentration in crude oil are well developed nowadays. They can be divided into two classes roughly: offline measurement and online measurement. The former contains Distillation [10], Near-infrared (NIR) [11], Ultrasound [5,12], and Coplanar Waveguide (CPW) [13]. The common drawback of these methods is manual sampling, which is always coupled with randomness and failure to indicate the change of oil concentration timely. It is even more inappropriate to apply these measurement methods to dispersive oil wells, especially in poor weather conditions. The developments of the methods are greatly restrained for being unable to meet the needs of automatic production and management. The online measurements represented by Hydrometer [14], γ-ray [15,16], Capacitance [1,17,18,19], and Microwave [20] methods behave much better in this aspect. However, these methods have their own weaknesses. For example, Hydrometer method has the problem of low accuracy for sand-containing oil. Furthermore, the hydrometer installation is very rigorous for being sensitive to the external environment. These problems can be very difficult to overcome. γ-ray method works based on the difference of γ-ray absorption between oil and water. The relatively small difference determines that the water content measurement will inevitably suffer from low precision. Additionally, this method has other disadvantages of radiation pollution, high cost, and poor accessibility. Oil content measurement based on Capacitance method takes the advantage of significant permittivity difference between water and oil. In the past two decades, Electrical Capacitance Tomography (ECT) has been developed, aiming for multi-phase flow measurement. It is useful in monitoring the parameters of the non-uniform flows including the Water-in-Liquid Ratio (WLR), the thickness of liquid layer and the position of the gas core [19]. However, the lack of flexibility of the measurement range destines that it is only suitable for the oil field with oil content lower than 30%. As for the Microwave method, it is also not very popular for the reason of high cost, as well as inconvenient usage and maintenance.

With development of oil production and management, the demand for efficient salt and oil content measurement techniques is becoming more and more urgent [8,9,13]. For instance, improvement of the measurement efficiency makes it possible to shorten the logging period and assess the status of oil well timely. Increasing sensing accuracy can offer more reliable data to the management. To suit the needs of modern industrial automation, the online measurement of salt and oil content has to evolve as well. To meet this demand, this article proposed a novel sensing method of ternary solutions composed of water, oil and salt. Conductivity method and ultrasound method are adopted, respectively, for estimating the oil and salt concentration in the solution. Traditional multi-parameter measurement is usually fulfilled with the joint efforts of several sensors, which might be bulky, complicated and have high-power consumption. In order to overcome the shortcomings, multifunctional sensor based on cross sensitivity theory is applied by combining the two parts of transducers so that the measurement mission can be accomplished with fewer devices. It is theoretically possible that the input signals of the multifunctional sensor, coupling with signal reconstruction algorithm, would be calculated reversely to achieve the measurement online. The proposed method provides a very promising solution to the problems of precision, realtimeness, and automation, which are encountered by the existing methods.

The contributions of this paper mainly focus on the following aspects. First of all, a novel multifunctional sensor is designed and manufactured for concentration measurement of the ternary solution, which is made up with oil, salt, and water. Secondly, Simple Correlation Analysis (SCA) and Canonical Correlation Analysis (CCA) are applied to quantify the relationship between the input and output signals of the sensor for the purpose of verifying the rationality of the design. At last, a signal reconstruction method is applied to estimate the sensor inputs using the outputs, which proves the effectiveness of its usage.

The paper is organized as follows. In Section 2, we introduce the structure of the multifunctional sensor and the measurement principle of the solute concentration in the ternary solution. In Section 3, the experiment design and data acquisition are proposed. In Section 4, we formulate the derivation of Canonical Correlation Analysis. In Section 5.1 and Section 5.2, we present the analysis results for the sensor data based on SCA and CCA methods, respectively. In Section 6, a signal reconstruction method based on Support Vector Machine and the corresponding reconstructed results of the sensor are introduced. In Section 7, we give a conclusion about the experimental result of the multifunctional sensor.

## 2. Structure of the Multifunctional Sensor

The multifunctional sensor is designed to measure the salt and oil concentrations of a ternary solution online. Provided with accuracy and stability, it is able to measure several parameters simultaneously. With compact size, it also possesses excellent characters of corrosion resistance and long service life.

**Figure 1:**
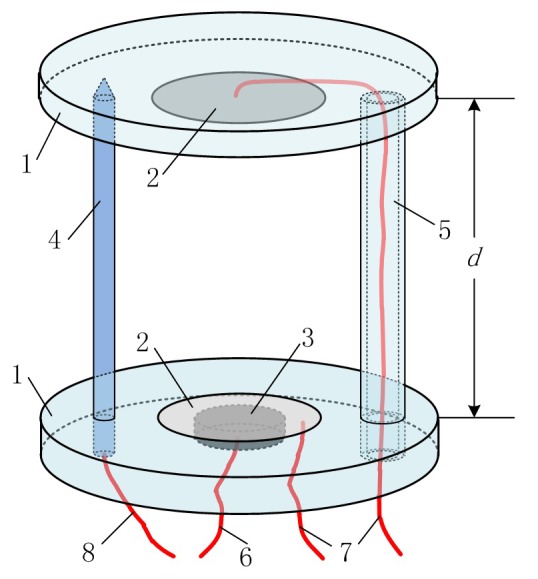
Sensor probe: (**1**) acrylic resin substrate; (**2**) stainless steel electrode; (**3**) piezoelectric transducer; (**4**) thermometer; (**5**) support beam; (**6**) ultrasonic transducer wire; (**7**) conductivity sensor wire; and (**8**) Thermometer wire.

The structure of multifunctional sensor is shown in Figure 1 and mainly consists of several parts: substrate (acrylic resin plate, 3 mm thick, 56 mm in diameter), conductivity electrodes (stainless steel, 0.5 mm thick, 25 mm in diameter), piezoelectric transducer (piezoelectric ceramic, 2 mm thick, 13 mm in diameter), thermometer (platinum resistance, 50 mm long, 4 mm in diameter), and support beam (acrylic resin plate, 45 mm long, 4 mm in inner diameter and 8 mm in outer diameter). The sensor has two functions: one is ultrasound-based velocimetry, and the other is conductivity sensing. The ultrasound transducer works in the mode of pulse-echo. By applying AC voltage to the piezoelectric (PZT) material (component 3), an ultrasound (2 MHz sine wave) will be generated from the bottom of the sensor. The wave will travel through the water and reach the upper plate (component 2). After rebound by the upper plate, it will finally return to the PZT and motivate AC voltage according to theory of inverse piezoelectric effects. Based on the output electrical signal, Time of Flight (TOF) could be calculated and recorded. Then the ultrasound speed can be achieved with the known length of wave path, 2d. The test of conductivity is enabled by using the stainless steel electrodes, which, shown as element 1 in Figure 1, are embedded in the acrylic resin substrate (component 1). If the electrodes are applied by a constant electric field, a stable current will occur between them. Thus, it is easy to obtain the resistance and the conductivity of the solution with the measurement of the voltage difference. Since the effects of temperature on ultrasound speed and solution conductivity should not be neglected, a thermometer is installed on the sensor. Considering the asymmetry of temperature, we chose a thermistor probe (component 4) and bolted it between the substrate, which is also used to stabilize and balance the sensor with the support beam (component 5).

The sensor is capable of measuring multiple parameters including ultrasound speed, conductivity and temperature. Based on the nonlinear relationship between the input parameters (salt concentration, oil concentration, and temperature) and output ones (ultrasound speed and solution conductivity), salt content and water content can be both estimated with a proper algorithm in a four-dimensional space. A distinctive feature of the multifunctional sensor is that the electrode plate (component 2), one part of the conductivity transducer, is also used as the reflector of ultrasound. In this way, the ultrasound sensing and conductivity sensing are aligned perfectly, making it a multifunctional sensor in a real sense. There are two important things to note about the usage of the multifunctional sensor, especially for the measurement of crude oil. One is the ultrasonic attenuation property. Ultrasound decays rapidly in the case that gas bubbles suspend in the oil, which means the multifunctional sensor cannot work under these circumstances. Thus, an online defoaming procedure such as heating, stirring, or defoamer addition must be conducted before the measurement. The other thing is that heterogeneous distribution of oil and water are very common in crude oil. This phenomenon limits the measurement reliability of both the conductivity sensor and the ultrasound sensor. To achieve credible results, the mixed solution needs to be homogenized by adding emulsifying agents, which could be easily implemented online.

### 2.1. Principle of Conductivity Measurement

**Figure 2:**
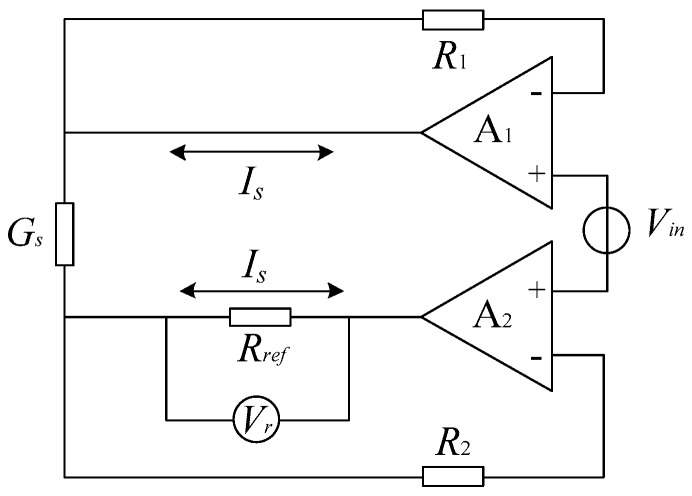
Equivalent circuit of conductivity measurement.

The equivalent circuit of conductivity sensing is shown in Figure 2. The input voltage $V_{in}$ provided by signal generator is a sinusoidal signal with frequency of 1 kHz and amplitude of 1 V. Because the input impedances of the amplifiers $A_{1}$ and $A_{2}$ are both extremely high, the currents flowing through the resistance $R_{1}$ and $R_{2}$ are close to zero approximately. Since $V_{in}$ is constant, the relationship between the solution conductance $G_{s}$ and the current $I_{Gs}$ flowing through it can be expressed by:

(1) $$I_{Gs}=V_{in}G_{s}.$$

According to the definition of conductivity, we can get the function of conductivity σ and $I_{Gs}$ shown as:

(2) $$I_{Gs}=\frac{V_{in}}{K}\sigma ,$$

with K standing for the conductivity cell constant. To facilitate the measurement, reference resistance $R_{ref}$ is plugged into the input of the amplifier $A_{2}$ to generate a stable current $I_{Gs}$. Because the current passing through the resistances $R_{1}$ and $R_{2}$ are near to zeros, there is little voltage on them. Based on the principle of dummy short, $V_{Gs}$, the voltage on $G_{s}$, is nearly equal to $V_{in}$, then we get:

(3) $$I_{Gs}=\frac{V_{r}}{R_{ref}}=\frac{V_{in}}{K}\sigma \approx \frac{V_{Gs}}{K}\sigma ,$$

namely:

(4) $$\sigma =\frac{K}{R_{ref}V_{ex}}V_{r}.$$

As shown in Equation (4), the conductivity of the solution is proportional to output voltage $V_{r}$. Since the parameters such as $R_{ref}$, K, and $V_{in}$ are constant for an identified solution, $V_{r}$, in practice, may reflect the variance of conductivity σ. Thus, we take the output voltage $V_{r}$, instead of σ, as one of the outputs of the multifunctional sensor in the following experiment.

### 2.2. Principle of Ultrasound Speed Measurement

**Figure 3:**
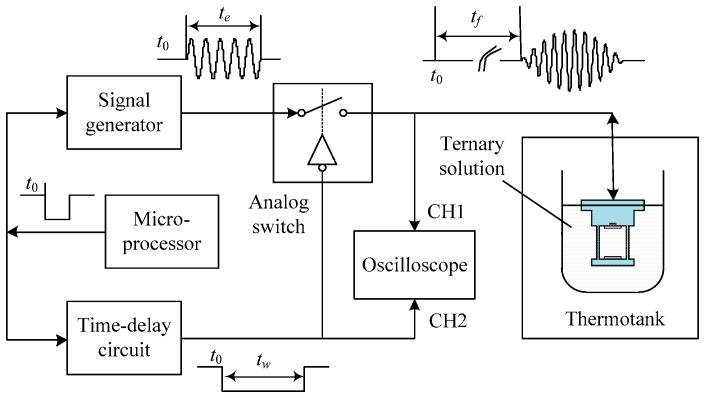
Schematic diagram of TOF measurement.

**Figure 4:**
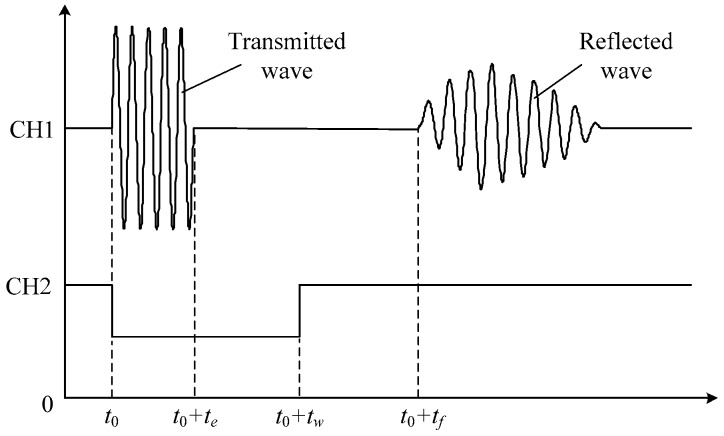
Sketch of transmitted and reflected waves.

As shown in Figure 1, the round-trip distance the ultrasound travels during the time from being generated to arriving at the receiver is fixed at 2d. Thus, the velocity $v_{s}$ and the time of flight $t_{f}$ conform to:

(5) tf=2dvs,

which means that $t_{f}$ is inverse proportional to $v_{s}$. For the reciprocal relation, we replace the velocity with TOF as the other output of the multifunctional sensor to demonstrate the variation caused by different concentration of the solutes. Figure 3 shows the measurement procedure of TOF. At time $t=t_{0}$, the signal generator controlled by the microprocessors generates a signal envelope as shown in Figure 4, which is composed of four cycles of sinusoidal waves with frequency of 2 MHz and amplitude of 10 V. Simultaneously, time-delay circuit is also controlled to initiate its timer and keeps the analog switch closing until $t_{w}$. During this period, the input signals can reach the piezoelectric material and excite the ultrasonic waves. At the moment $t=t_{w}$, the switch disconnects the path of input signals and then makes the channel CH1 of the oscilloscope receive the echo signals. To make it work, it has to be guaranteed that the delay time lasts longer than the duration of the input signals $t_{e}$, namely $t_{w}>t_{e}$. The ultrasound keeps bouncing back and forth between the emitter/receiver and the reflector until it vanishes. Thus, the echo, as a matter of fact, is a pulse train composed by a series of envelope signals. For the sake of accuracy, we regard the time that the receiver catches the first impulse among the echoes as the end of one test. By using level detection circuit, $t_{f}$, namely TOF, shown in Figure 4, can be finally measured and recorded. It is the time lag between emitting and receiving of the ultrasound, and needs to be recorded by oscilloscope manually.

## 3. Experiment

To simulate the crude oil containing salt and water, we choose a petroleum derivative, Hydraulan Dot4 braking fluid, for consideration of stability of the mixed solution. The braking fluid dissolves in water so that the solution made of it is homogeneous. All the solutes and solvents are miscible with each other requiring neither stirring nor an emulsifier. In this way, the bubbles accompanied with stirring will never appear, which would scatter and absorb ultrasound tremendously. The excellent dissolution makes sure that the ultrasound sensor works properly and the measurement precision can be improved correspondingly.

The first step of the experiment is preparing the gradient solutions. To simulate the actual condition under which it works, we mix water and salt according to the ratios of 96:4, 94:6, 92:8, 90:10, 88:12, 86:14, 84:16, 82:18 and 80:20. After dividing every solution into 9 units, we add the braking fluid of mass fraction of 20%, 27.5%, 35%, 42.5%, 50%, 57.5%, 65%, 72.5% and 80% with reference to the quality of the brine.

**Figure 5:**
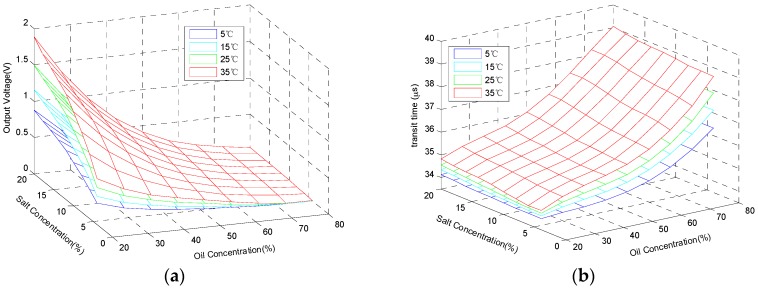
Experimental data: (**a**) Output voltage; and (**b**) Time of Flight.

The second step is collecting the data of the multifunctional sensor. As clear as the descriptions above, 81 samples of solutions are needed throughout the experiment. The procedures of measurement for each sample are the same: put the sample into a thermostat, then record the output voltage and TOF in the condition that the temperature of the sample is steady at 5 °C, 15 °C, 25 °C and 35 °C. The final data are drawn in Figure 5. In total, there are 5 parameters of the sensor inputs and outputs, so it is impossible to display all of them in one figure visually. Via dimension reduction, the parameters are divided into two groups and described in Figure 5a,b. Furthermore, therein, Figure 5a shows 4 parameters: salt concentration, oil concentration, solution temperature, and output voltage, and Figure 5b demonstrates the other composition of parameters: salt concentration, oil concentration, solution temperature, and TOF.

## 4. Canonical Correlation Analysis (CCA)

In order to illustrate the internal correlations among the parameters of the presented multifunctional sensor, and then demonstrate the correctness of its design and usage theoretically, we conduct a correlation analysis concerning the recorded data. Canonical Correlation Analysis (CCA) [21], similar with Principal Component Analysis (PCA) [22], selects several representative variables from the sets to compose synthesized indicators called canonical variables. Studying the correlation among these canonical variables rather than the groups of the original ones makes more sense and will help us take more rational decisions [21,23].

Given two sets of variables, x and y, with the dimension of $p_{1}$ and $p_{2}$ under the condition of $p_{1}\leq p_{2}$, we can get:

(6) $$\{\begin{array}{c} z=\left[\begin{array}{c} x\\ y \end{array}\right],\;E\left[z\right]=\left[\begin{array}{c} \mu_{1}\\ \mu_{2} \end{array}\right]\\ \Sigma =\mathrm{var}(z)=E\{(z-E[z]){\left(z-E[z]\right)}^{T}\}=\left[\begin{array}{cc} \Sigma_{11} & \Sigma_{12}\\ \Sigma_{21} & \Sigma_{22} \end{array}\right] \end{array}\;,$$

where Σ represents the variance matrix of z. $\Sigma_{11}$ and $\Sigma_{22}$ are, respectively, on behalf of those of x and y. $\Sigma_{12}$ stands for their covariance with the transpose expressed as $\Sigma_{21}$.

Define the matrixes, u and v, as the canonical variables of x and y with:

(7) $$\{\begin{array}{c} u=a^{T}x\\ v=b^{T}y \end{array},$$

where a and b stand for their eigenvector coefficients. As expected, the correlation coefficient is obtained based on:

(8) $$\mathrm{corr}(u,v)=\frac{a^{T}\Sigma_{12}b}{\sqrt{a^{T}\Sigma_{11}a}\sqrt{b^{T}\Sigma_{22}b}}.$$

Then we need to calculate the coefficients a and b by maximizing:

(9) $$J=\max(a^{T}\Sigma_{12}b).$$

Amplified or shrunk proportionally, a and b still conform to Equation (9), which means Equation (9) has infinite solutions. To avoid this, it is necessary to add constraints in Equation (13):

(10) $$\{\begin{array}{c} a^{T}\Sigma_{11}a=1\\ b^{T}\Sigma_{22}b=1 \end{array}.$$

The calculations of the coefficients could be achieved by constructing Lagrangian equation:

(11) $$L=a^{T}\Sigma_{12}b-\frac{\lambda }{2}\left(a^{T}\Sigma_{11}a-1\right)-\frac{\theta }{2}\left(b^{T}\Sigma_{22}b-1\right).$$

The eigenvalues of corr(u,v) are achieved in the form of:

(12) $$\lambda =\theta =a^{T}\Sigma_{12}b.$$

On the premise that $\Sigma_{11}$ and $\Sigma_{22}$ are invertible, Equation (12) may be simplified as:

(13) $$\{\begin{array}{c} \Sigma_{11}^{-1}\Sigma_{12}b=\lambda a\\ \Sigma_{22}^{-1}\Sigma_{21}a=\lambda b \end{array}.$$

Apparently, the coefficients a and b, as well as the canonical correlation, corr(u,v), can be easily obtained, as long as the maximum eigenvalue, $\lambda_{\max}$, is acquired. Suppose that λ is the maximum of all the eigenvalues, then the coefficients we just obtained from the calculations are actually $a_{1}$ and $b_{1}$, the eigenvector coefficients of λ. Then the equations:

(14) $$\{\begin{array}{c} u_{1}=a_{1}^{T}x\\ v_{1}=b_{1}^{T}y \end{array},$$

are established with $u_{1}$ and $v_{1}$ being the first set of canonical variables. λ is the canonical correlation of u and v we desire. In the same way, we can finally calculate all $p_{1}$ sets of canonical variables.

## 5. Correlation Analysis of Experimental Data

### 5.1. Simple Correlation Analysis

Based on the theory mentioned above, Simple Correlation Analysis, namely Pearson Product-moment Correlation Coefficient [24], is carried out for the data of multifunctional sensor. Firstly, regard sensor inputs such as oil concentration, salt concentration and temperature as the observable variable x. As for the observation variable y, it is composed of output voltage and TOF, namely the outputs of the sensor. By conducting the auto- and cross-correlation analysis, the simple coefficient of correlation shown in Table 1 is achieved. Owning smaller coefficients means that the two variables have less overlapping information. On the contrary, the ones with bigger coefficients have more things in common. Additionally, if the coefficient is greater than zero, there will be a positive correlation between the two variables, which means they will evolve in the same direction. On the other hand, if the coefficient is less than zero, the correlation is negative so that the variables will increase or decrease inversely.

As shown as the internal correlation coefficients of x, oil concentration, salt concentration, and temperature are all independent of each other. This matches up with the principle of experiment setting. The auto-correlation of y is actually used for indicating the relationship between output voltage and TOF, which is negative and significant.

More importantly, Table 1 gives the correlation between the inputs and outputs of the sensor. Obviously, output voltage is closely related to all of the input variables. Its positive correlations with salt concentration and temperature as well as its negative correlation with oil concentration can be explained from the physical point of view. In the section of conductivity measurement, the positive and the negative ions will keep moving oppositely to create a current in the electric field. The generated potential difference is the output voltage we mentioned above, which is proportional to the conductivity of the solution. The quantity of conductivity is up to the migration rates of the ions. If salt content increases proportionally, the carriers’ concentration will increase correspondingly resulted in the rising of conductivity and output voltage. On the other hand, increase of oil concentration leads to decrease of the carriers. In the macroscopic view, resistance to the motions of ions becomes larger, which will guide to the reduction of conductivity and output voltage. Accompanied with increasing of temperature, solution viscosity drops and reduces the resistance of ion migration. As a result, conductivity and output voltage increase along with the temperature. As for output voltage, the weights of temperature, salt and oil concentrations can be demonstrated with the absolute values of their correlation coefficients. Analyzing the data listed in Table 1, we can draw the conclusion that oil content plays a primary role in determining output voltage, and the influence of salt content and temperature is relatively small.

As a mechanical wave, ultrasound interacts with the media around it and its propagation character is closely related to media’s physicochemical properties. The speed of ultrasound is relevant to the adiabatic compressibility and density of the solution, which can be expressed by:

(15) $$\frac{1}{v_{\mathrm{mix}}^{2}}=\sum_{i=1}^{n}x_{i}\rho_{i}K_{i}$$

where, $v_{\mathrm{mix}}$ is the velocity of ultrasound travelling in water, and $x_{i}$ stands for mass fraction of Solute i. $\rho_{i}$ and $K_{i}$ represent its density and isothermal compressibility, which is equal to the reciprocal of elasticity modulus numerically. On the premise that there is no other fluid source introduced into the solution, the wave equation of ultrasound will be defined with:

(16) $$\frac{1}{v_{i}^{2}}=\rho_{i}K_{i}$$

where $v_{i}$ is the velocity of ultrasound while travelling in solution i. For the ternary solution used in this experiment, Equation (15) can be transformed into:

(17) $$\frac{1}{v_{\mathrm{mix}}^{2}}=\frac{x_{s}}{v_{s}^{2}}+\frac{x_{o}}{v_{o}^{2}}$$

where $v_{s}$ and $v_{o}$ stand for the ultrasound speed in oil and brine, whose mass fractions, represented as $x_{s}$ and $x_{o}$, are constant for a certain solution. Known from Equation (17), $v_{\mathrm{mix}}$ varies with $x_{s}$ and $x_{o}$: increasing of $x_{s}$ and $x_{o}$ leads to the decreasing of $v_{\mathrm{mix}}$, and then extension of TOF. It means TOF is positively associated with oil and salt concentration. This conclusion is also supported by the data in Table 1: the correlation coefficient between oil concentration and TOF is 0.8371 and that between salt concentration and TOF is 0.0990. The data also demonstrate that oil concentration take the lead in effecting TOF.

TOF, as shown in Table 1, is also related to temperature manifesting that the higher the temperature is, the longer it takes for ultrasound wave traveling between the electrode plates. This phenomenon can be explained by the temperature property of ternary solution. Compressibility coefficient $K_{0}$ and density $\rho_{0}$ of the solution vary as:

(18) $$K=K_{0}(1+\varepsilon \Delta T)$$

(19) $$\rho =\rho_{0}(1+\alpha \Delta T+\beta \Delta T^{2})$$

responding to the variation of the temperature. ΔT is temperature difference. ε, α and β are all the coefficients and usually treated as constants for a stable solution. From Equation (16), the ultrasound velocity can be derived as:

(20) $$v^{2}=\frac{v_{0}^{2}}{\left(1+\varepsilon \Delta T)(1+\alpha \Delta T+\beta \Delta T^{2}\right)}$$

where $v_{o}$ represents the velocity at temperature of $T_{0}$. Obviously, when the temperature rises, the velocity would be falling and lead to the increasing of TOF. The positive correlation between TOF and temperature matches the result obtained from Simple Correlation Analysis. The correlation coefficient of 0.4350 also demonstrates the significance of the influence on TOF by temperature.

### 5.2. Canonical Correlation Analysis

For the interaction between the variables, simple correlation coefficients are all for reference only, incapable of demonstrating the actual relationship from a unitary perspective. Canonical Correlation Analysis, on the contrary, is able to extract the representative aggregate variable from the sets and maximize the overall correlations among them. By dividing the experiment data into two groups of variables, input and output, the results of Canonical Correlation Analysis can be obtained as shown in Table 2.

The canonical correlations of the pairs of canonical variates are 0.963 and 0.776. The first pair of variates, a linear combination of input measurements and that of output measurements, has a correlation coefficient of 0.963. The second pair has a correlation coefficient of 0.776. Each subsequent pair of canonical variates is less correlated. The corresponding eigenvalues can be calculated by:

(21) $$E_{i}=C_{i}^{2}/\left(1-C_{i}^{2}\right)$$

where $C_{i}$ is the canonical correlation coefficient with the eigenvalue of $E_{i}$. i belongs to $\left[1,p_{1}\right]$, and for the data set of the proposed sensor, i=1,2. Clearly, the coefficients of Canonical Correlation Analysis are generally greater than those of Simple Correlation Analysis, which illustrates the distinction between them.

Since the above results are obtained from the experiment data, a null hypothesis of chi-square test is applied to the set of roots. The null hypothesis is that all of the correlations associated with the roots in the given set are equal to zero in the population. By testing these different sets of roots, we are determining how many dimensions are required to describe the relationship between the two groups of variables. Because each root in Table 2 is less informative than the one before it, unnecessary dimensions will be associated with the smallest eigenvalues. It is possible to pick out the roots that can describe the relationship between the two groups of variables without losing too much information. Thus, we start our test with the full set of roots and then test subsets generated by omitting the greatest root in the previous set. Then we repeat the procedure until there is only one root left.

Here, Wilk’s Lambda test statistic is used for testing the null hypothesis that the given canonical correlation and all smaller ones are equal to zero in the population. Each value can be calculated as the product of the values of (1-canonical correlation^2^) for the set of canonical correlations being tested, namely:

(22) $$S_{i}=\prod_{i}^{2}\left(1-C_{i}^{2}\right).$$

As can be seen, the smaller the value of Wilk’s Lambda, the greater the contribution offered by the canonical correlation coefficient. Thus, the contribution of the first coefficient is greater than that of the second one. Significance Level is the *p*-value associated with the F value of a given test statistic. The null hypothesis of the two sets of variables having no relationship is evaluated with regard to this *p*-value. For a given alpha level, such as 0.05, if the *p*-value is less than alpha, the null hypothesis is rejected. If not, then we fail to reject the null hypothesis. As shown in Table 2, the *p*-values corresponding to the two canonical correlation coefficients are both 0.000. It means that the null hypothesis is invalid and the coefficients are statistically significant. In conclusion, there is an obvious linear interrelationship between the inputs and outputs of the multifunctional sensor, whose correlation study can be converted to the correlation analysis of their canonical variates.

Table 3 and Table 4 present the input and output signal set of the sensor, respectively, including Raw Canonical Variable Coefficients (RCVC) and Standardized Canonical Variable Coefficients (SCVC), which can be abbreviated as raw coefficients and standardized coefficients. The former defines the linear relationship between the variables in the given group and the canonical ones. They can be interpreted in the same manner as regression coefficients, assuming the canonical variates as the outcome variables. When the variables in the model have very different standard deviations, RCVC do not allow for easier comparisons among the variables. This problem induces the presence of SCVC. If all of the variables in the analysis are rescaled to have a mean of zero and a standard deviation of 1, the coefficients generating the canonical variates would indicate how a one standard deviation increase in the variable would change the variates. SCVC are interpreted in a manner analogous to interpreting standardized regression coefficients. Lack of consistent dimension of sensor inputs and outputs, it would be better to adopt SCVC in such case. With the information shown in Table 3 and Table 4, the first canonical variates of input and output sets, $U_{1}$ and $V_{1}$, may be acquired as:

(23) $$\{\begin{array}{c} U_{1}=0.966x_{1}^{*}-0.004x_{2}^{*}+0.258x_{3}^{*}\\ V_{1}=-0.337y_{1}^{*}+0.792y_{2}^{*} \end{array}$$

where, $x_{1}^{*}$, $x_{2}^{*}$ and $x_{3}^{*}$ stand for the standardized variables of oil concentration, salt concentration, and temperature. $y_{1}^{*}$ and $y_{2}^{*}$ are defined as those of output voltage and TOF. In the same way, the second canonical variates, $U_{2}$ and $V_{2}$, can also be determined as:

(24) $$\{\begin{array}{c} U_{2}=-0.229x_{1}^{*}+0.448x_{2}^{*}+0.864x_{3}^{*}\\ V_{2}=1.094y_{1}^{*}+0.826y_{2}^{*} \end{array}$$

According to Equations (23) and (24), $U_{1}$ is mainly on behalf of oil concentration, and TOF is the main component of $V_{1}$. While, $U_{2}$ stands for salt concentration and temperature chiefly, and $V_{2}$ represents for all output variates. We can draw the conclusion that the first canonical variate does not play an important role in explaining salt concentration. Furthermore, both the first and second variates are closely related to every set of signals.

In order to explain to what extent the canonical variates expressing the observational ones, the computations of canonical loading coefficient and cross loading coefficient are carried out. The former, also known as coefficient of structural relationship, is the pair-wise correlation coefficient between the canonical variates and the observational ones. It is the complement to canonical variate coefficient and can be considered as the total effect observational variates caused by the canonical ones, while cross loading coefficient is the pair-wise correlation coefficient between the canonical variates and the other set of observational variates. The purpose of studying this coefficient is to construct the relationship between the sets. Table 5 lists these two kinds of coefficients of the sensor inputs. Since the input signals are mutual independent, canonical loading coefficients are identical to those of canonical variates. Apparently, canonical variates corresponding to sensor inputs mainly explain the variables of oil concentration and temperature. Canonical and cross loading coefficients of sensor outputs are shown in Table 6. Judging from the values, Equations (23) and (24) can adequately represent the output variables of the multifunctional sensor. In conclusion, correlation analysis of observational variates is equivalent to Canonical Correlation Analysis. Using correlation analysis of observational variates, instead of CCA, would make the correlation study between the inputs and outputs become more reasonable and intuitive.

## 6. Signal Reconstruction

**Figure 6:**
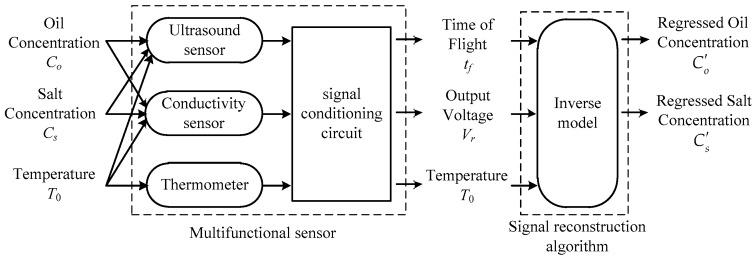
Schematic structure of multifunctional sensing technique.

In general, the multifunctional sensing technique is composed of two procedures, sensing and reconstructing. The former takes charge of multiple variable detection with sensitive components based on their crossing sensitivity properties, and the latter is responsible for regressing the measured variables by using corresponding algorithm. The signal sensing and reconstructing procedure is shown in Figure 6, where Oil Concentration, Salt Concentration, and Temperature are the physical quantities under measurement, Time of Flight and Output Voltage are the sensor output signals, while Regressed Oil Concentration and Regressed Salt Concentration are the estimation of the measured quantities that can be obtained through the signal reconstruction algorithm.

Accompanying the development of multifunctional sensors, signal reconstruction algorithm is well studied. Empirical Risk Minimization (ERM) principle is popular in these methods, which ensures the actual risk close to the value of empirical risk when the sample data set is large. The signal reconstruction is usually a high-dimensional signal processing problem, while, the sample data set obtained from the experiment, on the contrary, is relatively small. In this case, minimizing the empirical risk cannot guarantee a small value of actual risk, and thus lead to the overfitting and poor generalization capabilities [25]. Support Vector Machine (SVM) could provide powerful and efficient tools that are capable of dealing with the small sample size problem and theoretical bounds on the generalization error through replacing ERM principle with Structural Risk Minimization (SRM) principle, which defines a tradeoff between the quality of the approximation of given data set and the complexity of approximating function, motivated by statistical learning theory [26].

Considering the outstanding performance of SVM in machine learning, we applied it to the reconstruction of the proposed multifunctional sensor. As shown in Figure 5, there are 81 samples of solutions included in the experiment. In order to verify the effectiveness of both the sensor and the reconstruction algorithm, these samples are divided into two groups randomly. One group is the training data set comprising 61 samples. The other, the testing data set, is made up of the remaining samples, which are used to evaluate the model constructed by SVM algorithm with the training data.

**Figure 7:**
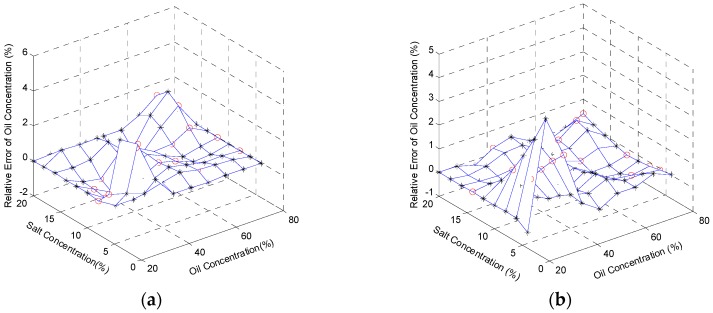

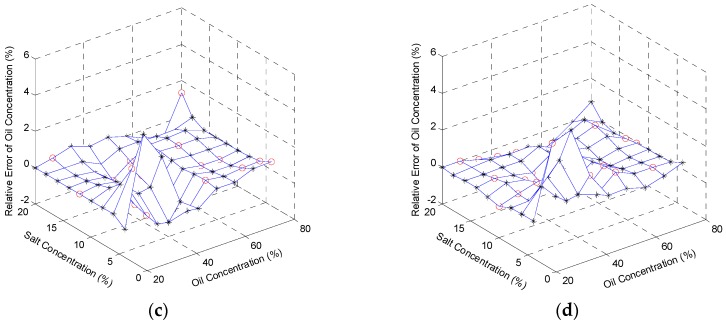
Relative errors of reconstructed oil concentrations at: (**a**) 5 °C; (**b**) 15 °C; (**c**) 25 °C; and (**d**) 35 °C. Black stars (*) stand for the relative errors of the training data, and red circles (o) represent those of the testing data.

**Figure 8:**
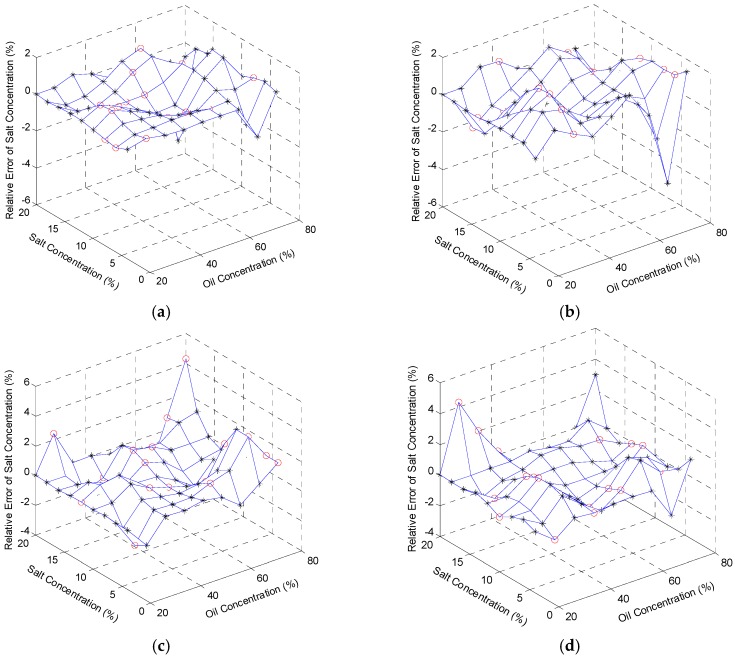
Relative error of reconstructed salt concentration at: (**a**) 5 °C; (**b**) 15 °C; (**c**) 25 °C; and (**d**) 35 °C. Black stars (*) stand for the relative errors of the training data, and red circles (o) represent those of the testing data.

The oil concentrations, at 5 °C, 15 °C, 25 °C, and 35 °C, were regressed based on the reconstruction algorithm with the relative errors shown in Figure 7a–d. The maximum values of the errors are respectively 4.13%, 4.21%, 4.42%, and 4.26%. In the same way, the salt concentrations were also regressed based on the reconstruction algorithm at 5 °C, 15 °C, 25 °C, and 35 °C. The figures of the relative errors are shown in Figure 8a–d, respectively, with the maximum values of the errors, 4.63%, 4.27%, 4.32%, and 4.27%. In general, the reconstructed signals are relatively precise and satisfactory. The results demonstrate not only the correlation between sensor inputs (oil concentration, salt concentration, and temperature) and sensor outputs (output voltage and TOF), but also the feasibility of the multifunctional sensor used in content detection of the ternary solution.

## 7. Conclusions

To meet the requirement of oil and salt content test in the process of production, storage, and refinement of crude oil, we presented a multifunctional sensor integrated with conductivity and ultrasound transducers. We also prepared ternary solutions composed of water, oil, and salt, and then carried out the gradient experiments. After recording and processing the experimental data, we came to several conclusions.

Firstly, the higher the solution temperature is, the slower the ultrasound travels in it. The increase of oil and salt contents will lead to reduced wave velocity. Compared with salt content, oil content is more dominant in the effect.

Secondly, under the condition of oil and salt contents being constant, solution conductivity will rise along with its temperature. Given the temperature and the salt content, the changing trend of the conductivity will be opposite to that of oil content. For the fixed temperature and oil content, the increasing salt content will improve the conductivity of ternary solutions. Furthermore, the improvement is more significant than those caused by the variations of oil content and temperature.

Finally, we conducted correlation analysis for the multifunctional sensor input set (salt concentration, oil concentration and temperature) and output one (output voltage and TOF). From the perspectives of Simple Correlation Analysis and Canonical Correlation Analysis, the relationships between input variates and output ones are established numerically. According to the calculated coefficients, we estimated the contributions of all the variables to the results, and then presented the variation tendency of the experimental data theoretically. The results indicated that there are essential connections between the inputs and outputs, which testified the correctness of the design of the multifunctional sensor and the reliability of the achieved results. Taking advantage of the conclusion, we applied a signal reconstruction method, Support Vector Machine, to the multifunctional sensor for the purpose of data training and testing. The reconstructed results indicate that the multifunctional sensor is capable of detecting the oil and salt concentrations in the ternary solution with high accuracy. The performance of sensing and reconstructing procedure can both meet the requirements of the application, which proves that online estimations of concentrations for ternary solution, even for crude oil, can be theoretically realized based on the modeling of this multifunctional sensor.

## Figures and Tables

**Table 1:** Simple correlation coefficients of input and output variables.

	Voltage	Transit Time	Oil Concentration	Salt Concentration	Temperature
Voltage	1.0000	−0.4864	−0.7951	0.2430	0.2845
Transit Time	−0.4864	1.0000	0.8371	0.0990	0.4350
Oil Concentration	−0.7951	0.8371	1.0000	0.0000	0.0000
Salt Concentration	0.2430	0.0990	0.0000	1.0000	0.0000
Temperature	0.2845	0.4350	0.0000	0.0000	1.0000

**Table 2:** Canonical Correlation Analysis and Test Results.

Roots	Canonical Correlation	Eigen Value	Significance Level
1	0.963	12.768	0.000
2	0.776	1.514	0.000

**Table 3:** Canonical variate coefficients of input set.

	Standardized Canonical Coefficients		Raw Canonical Coefficients	
	1st Can. Var.	2nd Can. Var.	1st Can. Var.	2nd Can. Var.
Oil Concentration	0.966	−0.229	0.050	−0.012
Salt Concentration	−0.004	0.448	−0.001	0.087
Temperature	0.258	0.864	0.023	0.077

**Table 4:** Canonical variate coefficients of output set.

	Standardized Canonical Coefficients		Raw Canonical Coefficients	
	1st Can. Var.	2nd Can. Var.	1st Can. Var.	2nd Can. Var.
Voltage	−0.337	1.094	−0.920	2.986
Transit Time	0.792	0.826	0.648	0.676

**Table 5:** Loading coefficients of input set.

	Canonical Loading Coefficients		Cross Loading Coefficients	
	$U_{1}$	$U_{2}$	$V_{1}$	$V_{2}$
Oil Concentration	0.966	−0.229	0.931	−0.178
Salt Concentration	−0.004	0.448	−0.004	0.348
Temperature	0.258	0.864	0.249	0.671

**Table 6:** Loading coefficients of output set.

	Canonical Loading Coefficients		Cross Loading Coefficients	
	$V_{1}$	$V_{2}$	$U_{1}$	$U_{2}$
Voltage	−0.722	0.692	−0.696	0.537
Transit Time	0.956	0.294	0.921	0.229

## Abbreviations

The following abbreviations are used in this manuscript:

ECT	Electrical Capacitance Tomography
WLR	Water-in-Liquid Ratio
SCA	Simple Correlation Analysis
CCA	Canonical Correlation Analysis
PZT	Piezoelectric
TOF	Time of Flight
PCA	Principal Component Analysis
RCVC	Raw Canonical Variable Coefficients
SCVC	Standardized Canonical Variable Coefficients
ERM	Empirical Risk Minimization
SVM	Support Vector Machine
SRM	Structural Risk Minimization
